# Paramedic attitudes towards prehospital spinal care: a cross-sectional survey

**DOI:** 10.1186/s12873-022-00717-2

**Published:** 2022-09-20

**Authors:** Neil McDonald, Dean Kriellaars, Rob T. Pryce

**Affiliations:** 1 Applied Health Sciences, University of Manitoba, Winnipeg, MB Canada; 2 Winnipeg Fire Paramedic Service, 2546 McPhillips St, Winnipeg, Manitoba R2P 2T2 Canada; 3 College of Rehabilitation Sciences, Rady Faculty of Health Sciences University of Manitoba, 771 Mc Dermot Ave, Winnipeg, Manitoba R3E 0T6 Canada; 4 Department of Kinesiology and Applied Health, Gupta Faculty of Kinesiology University of Winnipeg, 400 Spence St, Winnipeg, Manitoba R3B 2E9 Canada

**Keywords:** Emergency medical services, Paramedic, Prehospital, Spinal injuries, Survey

## Abstract

**Background:**

The optimal application of spinal motion restriction (SMR) in the prehospital setting continues to be debated. Few studies have examined how changing guidelines have been received and interpreted by emergency medical services (EMS) personnel. This study surveys paramedics’ attitudes, observations, and self-reported practices around the treatment of potential spine injuries in the prehospital setting.

**Methods:**

This was a cross-sectional survey of a North American EMS agency. After development and piloting, the final version of the survey contained four sections covering attitudes towards 1) general practice, 2) specific techniques, 3) assessment protocols, and 4) mechanisms of injury (MOI). Questions used Likert-scale, multiple-choice, yes/no, and free-text responses. Exploratory factor analysis (EFA) was used to identify latent constructs within responses, and factor scores were analyzed by ordinal logistic regression for associations with demographic characteristics (including qualification level, gender, and years of experience). MOI evaluations were assessed for inter-rater reliability (Fleiss’ kappa). Inductive qualitative content analysis, following Elo & Kyngäs (2008), was used to examine free-text responses.

**Results:**

Two hundred twenty responses were received (36% of staff). Raw results indicated that respondents felt that SMR was seen as less important than in the past, that they were treating fewer patients than previously, and that they follow protocol in most situations. The EFA identified two factors: one (Judging MOIs) captured paramedics’ estimation that the presented MOI could potentially cause a spine injury, and another (Treatment Value) reflected respondents’ composite view of the effectiveness, importance, and applicability of SMR. Respondents with advanced life support (ALS) qualification were more likely to be skeptical of the value of SMR compared to those at the basic life support (BLS) level (OR: 2.40, 95%CI: 1.21–4.76, *p* = 0.01). Overall, respondents showed fair agreement in the evaluation of MOIs (k = 0.31, 95%CI: 0.09–0.49). Content analysis identified tension expressed by respondents between SMR-as-directed and SMR-as-applied.

**Conclusion:**

Results of this survey show that EMS personnel are skeptical of many elements of SMR but use various strategies to balance protocol adherence with optimizing patient care. While identifying several areas for future research, these findings argue for incorporating provider feedback and judgement into future guideline revision.

**Supplementary Information:**

The online version contains supplementary material available at 10.1186/s12873-022-00717-2.

## Background

Major changes over the last decade in the standard of care for treating potential spine injuries in the prehospital setting have been described as a paradigm shift [1]. These changes have occurred across international jurisdictions, and new options for treatment allow greater flexibility than previous guidelines [2–6]. However, although the general principle of reducing movement has been widely endorsed, prehospital treatment recommendations falling under the heading of spinal motion restriction (SMR) still show significant differences. Some, for example, recommend the cervical collar as a critical component of care [7]; others recommend against it, propose a soft (as opposed to rigid) alternative, or forego its use in some situations [8–12]. This and similar questions continue to be debated [13–15].

Few studies have examined how emergency medical services (EMS) personnel have responded to these changes. A recent survey from Norway evaluated the use of a new national prehospital spinal treatment guideline, but was focused on implementation, not attitudes towards practice [16]. Other available research shows that emergency medical technicians (EMTs) in the United States believed that the prior practice of spinal immobilization (SI) was generally over-used, and that those with advanced life support (ALS) qualifications in particular viewed it as often unnecessary or not optimal in certain cases [1, 17, 18]. Limited research on SMR across countries demonstrates that EMTs and paramedics feel empowered and positive towards what are seen as progressive advances [1, 11].

In the context of evolving guidelines and limited information on provider attitudes, documented practice appears to vary more than might be explained by protocol changes. One study, focusing on geriatric patients with confirmed spinal injuries within a single service, reported that the number of patients who received no prehospital SMR rose from 15.5% to 31.6% after the transition to SMR protocols [19]. Another, assessing the shift to soft-collar use in emergency departments (in areas where local EMS used rigid collars), observed that up to one-third of trauma patients met criteria for spinal precautions but received none of any kind in both the hospital and prehospital settings [20]. Auditing the implementation of a prehospital selective immobilization protocol, Domeier *et. al.* found substantial lack of agreement among practitioners in the estimation of trauma with the potential to cause a spine injury [21]. This study concluded that up to 25% of patients did not receive an assessment in cases where the authors judged that the mechanism of injury (MOI) should clearly or possibly have triggered one. Other studies have documented rates of under- and over-treatment compared to assessment results ranging from 5 to 29% [22–24].

There is growing recognition that prehospital guidelines require the input and feedback from end users to ensure their applicability [25]. This work is informed by research into decision-making by EMS personnel and the role of clinical decision rules in prehospital and emergency settings [26–30]. As spinal-treatment guidelines continue to evolve, knowledge of how protocols are interpreted and applied in the field will be required to optimize patient care. Despite the relevance of best practices in trauma care across international jurisdictions, this area has received little attention. No study has used multiple methods to explore beliefs of EMS personnel around current procedures in the context of an evolving standard for spinal care. To fill this gap, this study surveys paramedics’ attitudes, observations, and self-reported practices around the treatment of potential spine injuries in the prehospital setting. In addition to describing survey findings, it specifically aims to analyze response data for latent constructs and insights, associations among results and provider characteristics, and provider agreement in areas of practice drawn from prior research.

## Methods

Reporting of this study conforms to the “Checklist for Reporting Of Survey Studies” (CROSS) guideline [31].

This was a cross-sectional survey of a single EMS agency located in central Canada. A draft survey was developed in consultation with local practitioners (*n* = 16) using a Delphi process modified to start with candidate questions informed by existing studies [32, 33]. This version was tested on a sample of EMS personnel at an international paramedic conference (*n* = 39). This process informed revisions to the final version, available as Additional file 1. The survey was organized in four sections:

1. Attitudes towards practice in general (nine questions, including six Likert-scale, two multiple-choice with free-text options, and one free-text)
2. Attitudes towards specific techniques and practices (13 questions, including 10 Likert-scale and three multiple-choice, one with a free-text option)
3. Attitudes towards spinal assessment protocols (six questions, including four Likert-scale and two multiple-choice with free-text options)
4. Judging MOIs (13 questions, including 12 yes/no choices and one multiple-choice with a free-text option)

This survey was disseminated within the Winnipeg Fire Paramedic Service (WFPS) in Winnipeg, Manitoba, Canada, with the cooperation of senior leadership and labour groups. No one outside the study team and development process had input into survey content. The WFPS responds to all 911 activations within a city of approximately 750,000. Using a tiered response model, it handles over 80,000 medical calls per year with basic life support (BLS) first response, and mixed BLS and advanced life support (ALS) transport. In common with many similar agencies, the WFPS has made several revisions to guidelines for the treatment of potential spine injuries. Most notably, it implemented a selective immobilization protocol based on the NEXUS criteria and similar to other prehospital algorithms in March 2009 [21, 34, 35]. As well, it adopted SMR treatment options in April 2016, which allowed for treatment with only a cervical collar in low-risk cases (as defined by patients who were ambulatory prior to EMS arrival) [36].

The survey was open to all licensed BLS and ALS providers within the service (*n* = 615 at the time of the survey, all of whom are termed “paramedics” under national certification guidelines). The survey was hosted on a commercial online platform (SurveyMonkey Inc, San Meteo, CA, www.surveymonkey.com). Information about the survey was distributed via memos and posters within the workplace; participation was anonymous and multiple responses were disabled by the survey platform. The target sample size was set at 200 to reflect a suggested minimum (*n* = 180, plus leeway for missing data) for exploratory factor analysis (EFA) with a survey of this structure [37]. The survey period started in December 2020, and a participation reminder was sent after one month; it was closed in April 2021 after reaching the target sample size. Distribution of the survey was approved by the University of Manitoba Health Research Ethics Board, HS22960 (H2019:252).

### Data analysis

#### Exploratory factor analysis

Although this survey was organized in sections that reflect topic areas drawn from prior research, it is unknown whether these correspond to how paramedics conceive the subject. In the absence of any previously validated survey instrument or established knowledge domains, EFA was conducted to identify potential latent constructs related to paramedic attitudes towards spinal care. Principal axis factoring and oblique rotation (direct oblimin) were used to identify factors. Extraction was determined by Cattell’s criterion (excluding the inflection point on the graph of eigenvalues). Factor loadings above 0.3 were included to set a low threshold for identifying contributing variables. On analysis, the Kaiser–Meyer–Olkin measure of sampling adequacy was 0.65, and Bartlett’s test of sphericity was significant at < 0.0001, x^2^(190) = 685.7. Summed factor scores were described for all participants by median, interquartile range (IQR), and range. Among identified factors, included variables were assessed for internal consistency using McDonald’s omega [38].

#### Demographic characteristics

A cumulative-odds ordinal logistic regression with proportional odds was run to determine the effect of demographic characteristics on factor scores. Characteristics included qualification level (ALS versus BLS), years of experience (greater than versus less than or equal to 10 years), and gender (woman versus man). The covariate, years of experience, was included to reflect the common finding that experience influences practitioners’ decision making [26–28]; the threshold of 10 years was informed by pilot results that demonstrated higher levels of participation among very experienced providers. To facilitate analysis, factor scores were partitioned into three, four, and five levels and tested for model fit. Based on these results, the final analysis used three levels for Judging MOIs and four levels for Treatment Value. Given the exploratory approach to the analysis, all Likert-scale questions included in the EFA but not contained within identified factors were analyzed on an individual basis by the same method using the levels from the question. Results for those questions with significant model fit are reported.

#### Inter-rater reliability of judging traumatic mechanisms of injury

Section 4 of the survey provided example MOIs and asked paramedics to categorize them as either having or not having potential to cause a spine injury. This choice reflects the decision point determining entry into prehospital selective treatment protocols, whether assumed or explicitly stated [35]. The wording in the survey mirrors the specific documentation requirements of the service. Inter-rater reliability was assessed with Fleiss’ kappa and applied overall, within demographic sub-groups identified above, and among groups of scenarios related by patient (geriatrics, pediatrics) or MOI type (falls, assaults, motor vehicle crashes [MVCs]) [39]. Two MOIs were included as calibration questions, with scenarios meant to be unambiguously positive or negative. In these questions, respondents showed near perfect agreement (k = 0.99).

All analysis was conducted in SPSS, version 28.0.1 (IBM Corporation; Armonk, New York USA) or R, version 4.0.5 (Foundation for Statistical Computing; Vienna, Austria). A threshold of alpha < 0.05 was considered statistically significant.

#### Content analysis

Seven questions in the survey allowed free-text responses. With no recent investigations of paramedics’ attitudes in this area, inductive qualitative content analysis was chosen as appropriate to explore and describe the phenomenon [40]. The analysis followed the process outlined by Elo and Kyngäs (2008) and informed by more recent methodological guidance [41, 42]. After preparation and in the process of de-contextualization, all responses were condensed and coded by two authors independently (NM, RP); during re-contextualization, codes were collected and inductively abstracted into sub-categories by both authors independently and then compared. Continuing abstraction occurred through discussion, during which both authors worked towards consensus by mutual questioning and reflection, iteratively reviewing and comparing the data and categories and maintaining congruence between levels of abstraction and degrees of interpretation [43, 44]. Results were discussed and reviewed with the third author [44]. The findings of the analysis describe the manifest content of the data with a low level of interpretation and high level of abstraction [42, 43].

#### Raw and missing data

Responses were received from 220 paramedics. This represents 36% of eligible staff, including those on leave. Raw data from Likert-scale and multiple-choice questions are presented in Additional file 2. Additional file 3 outlines free-text responses and corresponding sub-categories (with illustrative quotations) by each question. Notable findings from the undifferentiated data will be reported in the results.

Of 220 responses received, 179 completed all questions used in the quantitative analyses. Of the remaining 41, 23 were excluded outright because there were no responses beyond the initial demographics. Of the final 18, nine omitted all questions in Sect. 4, related to MOI. Therefore, all analyses involving Sect. 4 used 188 cases; remaining analyses used 197. Among these, there were 40 missing values (0.85% of the total); variable means (for continuous variables) or modes (for ordinal and categorical variables) were imputed in these cases.

## Results

Table**1** shows the demographic breakdown of the 197 respondents included in the main analysis.

**Table 1:** Characteristics of participants in a paramedic survey of spinal care

Characteristic	Number of respondents (percent)	Number in department (percent)
**Qualification Level**		
BLS	105 (53)	449 (73)
ALS	92 (47)	166 (27)
**Gender**		
Woman	62 (31)	133 (22)
Man	134 (68)	482 (78)
Transgender	0	-
Non-binary/non-conforming	0	-
Prefer not to respond	1 (0.5)	-
**Years of practice**		
< = 10	89 (45)	-
> 10	108 (55)	-
**Age**		
20–29	33 (17)	-
30–39	91 (46)	-
40–49	56 (28)	-
50–60	17 (9)	-

*BLS,* basic life support; *ALS,* advanced life support

Of note among the overall results, 70% of respondents felt they were treating “many” or “somewhat” fewer patients than in the past (question 1.7), a result mirrored by 71% who indicated that SMR is seen as “much” or “somewhat” less important than previously (question 1.8). A majority (74%) stated they apply the smallest available cervical collar most often (question 2.4). When asked whether they would either use precautions when not indicated by protocol (question 3.3) or not use them when they were indicated (question 3.5), majorities in both cases indicated “infrequently” or “never” (93% and 95%, respectively).

### Exploratory factor analysis

Two factors were identified during the EFA. Table 2 lists the items included in each factor, and Table 3 details factor eigenvalues, percent variance explained, and internal consistency. The first factor, labelled Judging MOIs, captures paramedics’ estimation that the presented MOI could potentially cause a spine injury. The score scale runs from 0 to 9, where high scores reflect more MOIs judged to have injury potential, and lower scores fewer. Among all respondents, the median factor score was 6 (IQR: 4–7, range 0–9). The second identified factor, termed Treatment Value, reflects respondents’ composite view of the effectiveness, importance, and applicability of SMR. Due to the scoring direction of individual questions, its maximum possible score [40] would indicate a high level of skepticism toward the value of treatment (or low level of endorsement), while the minimum [8] would indicate a low level of skepticism (or high level of endorsement). Overall, the median factor score was 26 (IQR: 24–29.75, range 19–37). Internal consistency of each factor was high (Judging MOIs, 0.77 and Treatment Value, 0.76). Item-drop and item-rest statistics were calculated for individual questions; item-rest correlations ranged from 0.31 – 0.58 and 0.23 – 0.61, for Judging MOIs and Treatment Value, respectively.

**Table 2:** Items included in factors identified by exploratory factor analysis in a paramedic spine survey

**Factor 1: Judging MOIs**		
**Question**	**Text (Yes/No response: Does this MOI have the potential to cause a spine injury?)**	**Factor loading**^1^
4.7	Elderly adult (> 65). Fall from standing. Laceration to the face. No loss of consciousness	0.68
4.3	Adult, assaulted. Punched in the face. No weapons used. Fell to the ground	0.68
4.8	Elderly adult (> 65), assaulted. Punched in the face. No weapons. Fell to the ground	0.64
4.11	Elderly adult (> 65). Syncopal episode. Fall from standing	0.64
4.5	Adult, tripped coming down stairs. Fell to the ground from one step	0.59
4.6	Adult, fall from standing. Laceration to the face. No loss of consciousness	0.54
4.12	Child (8 years old), fall from a slide onto grass, 2 m. Hit head. Unknown if there was a loss of consciousness	0.43
4.1	Child (7 years old), restrained on a booster seat on the driver’s side, rear. MVC while turning left. Hit by a vehicle travelling 40—50 km/hr on the passenger side. Moderate damage at point of impact. Front air-bags deployed. Windshield intact	0.42
4.9	Adult, restrained driver, MVC while turning left. Hit by a vehicle travelling 40—50 km/hr on the passenger side. Moderate damage at point of impact. Front air-bags deployed. Windshield intact	0.40
**Factor 2: Treatment Value**		
**Question**	**Text (Likert-scale response)**	**Factor loading**^1^
1.2	In your estimation, how often have you observed SMR ineffectively limit motion or cause more motion than no treatment or alternatives?	0.75
2.3	Among patients at risk for spine injury and in a cervical collar, how often do you observe patient movement that you feel could potentially cause further harm to their spine?	0.74
2.2	How often have you observed complications of a cervical collar resulting in more patient movement than no treatment or alternative / improvised treatment	0.67
1.1	In your opinion, how effectively does SMR as currently practiced limit patient motion?	0.67
1.3	Among patients at risk for spine injury and in SMR, how often do you observe patient motion that you feel could potentially cause further harm to their spine?	0.67
2.1	In your opinion, how effectively does a cervical collar restrict head motion in a potentially spine-injured patient?	0.51
1.8	Do you feel SMR is seen as less or more important than it was in the past?	0.38
3.2	In general and in your opinion, would you rate your service’s criteria for determining the need for spinal precaution as not restrictive enough (patients left untreated who need it) or too restrictive (too many patients treated who do not need it)?	0.38

Questions not included in factors: 1.4, 1.7, 2.5, 2.7, 2.8, 2.9, 2.10, 2.11, 2.12, 3.1, 3.3, 3.5, 4.4

1. Factor loadings > 0.3 reported in descending order

*MOI,* mechanism of injury; *MVC,* motor vehicle crash; *SMR,* spinal motion restriction

**Table 3:** Characteristics of factors identified by exploratory factor analysis in a paramedic spine survey

	Factor 1: Judging MOIs	Factor 2: Treatment Value
Eigenvalue	3.53	3.28
Percent variance explained	12	11
Internal consistency^1^	0.77	0.76
1. McDonald's Omega		

### Demographic characteristics

Table 4 presents the association of qualification level, years of experience, and gender with factor scores using ordinal logistic regression. ALS providers were significantly more likely to be more skeptical of treatment value than their BLS counterparts (OR 2.40, 95%CI: 1.21–4.76, *p* = 0.01), while men were less so than women (OR 0.53, 95%CI: 0.28–0.99, *p* = 0.05). Experience was not significantly associated with Treatment Value factor scores, and no demographic characteristic was associated with MOI factor scores.

**Table 4:** Qualification level, experience, and gender as predictors of factor scores^1^

Characteristic	Factor score—comparison	Factor score—reference	OR (95% CI)	p
***Factor 1: Judging MOIs (do the presented MOIs have the potential to cause a spine injury?)***				
ALS (ref BLS)	6 (4–7)	5 (3.5–7)	0.79 (0.40—1.54)	0.5
> 10 years exp. (ref < = 10 years)	6 (4–9)	5 (3–7)	1.72 (0.87—3.39)	0.1
Men (ref Women)	5 (3–7)	6 (4–7)	0.74 (0.41—1.34)	0.3
*Scoring range, direction: 0 (fewer with potential)—9 (more with potential)*				
*OR* > *1 means more likely to judge MOIs as potentially causing injury*				
*Likelihood ratio test (full model compared to intercept-only), x*^*2*^*(3)* = *3.52, p* = *0.3*				
***Factor 2: Treatment Value (composite view of the value of SMR)***				
ALS (ref BLS)	26.5 (24–29)	25 (22–27)	2.40 (1.21—4.76)	0.01
> 10 years exp. (ref < = 10 years)	26 (23–37)	25 (22–28)	1.25 (0.64—2.45)	0.5
Men (ref Women)	25 (22–28)	26 (24–29.75)	0.53 (0.28—0.99)	0.05
*Scoring range and direction: 8 (less skeptical)—40 (more skeptical)*				
*OR* > *1 means more likely to have a higher score*				
*Likelihood ratio test (full model compared to intercept-only), x*^*2*^*(3)* = *15.84, p* = *0.001*				

Questions with unique response patterns not included in identified factors were also analyzed in terms of demographic characteristics. Table 5 reports this analysis for the two questions whose overall model significantly predicted the dependent variable as compared to the intercept-only version. In question 2.7, ALS providers were significantly less likely to treat patients with isolated penetrating trauma than BLS providers, in accordance with local protocol (OR 0.10, 95% CI: 0.05–0.21, *p* < 0.001). In contrast, those with greater than 10 years of experience were more likely to say they would (OR 2.65, 95% CI: 1.41–4.99, *p* = 0.003). In question 1.7, ALS providers were much more likely than BLS to report the perception of treating fewer patients over time (OR 2.93, 95% CI: 1.58–5.43, *p* < 0.001). This result does not appear to reflect longer experience, as no association exists between rates of treatment and those with more than 10 years practice as compared to fewer (OR 1.33, 95% CI: 0.74–2.41, *p* = 0.3).

**Table 5:** Qualification level, experience, and gender as predictors of question scores^1^

Characteristic	Question score—comparison	Question score—reference	OR (95% CI)	p
***2.7 When treating a patient with isolated penetrating trauma to the head, neck, or torso,***				
***how often do you apply spinal precautions?***				
ALS (ref BLS)	2 (1–2)	3 (2–4)	0.10 (0.05—0.21)	< .001
> 10 years exp. (ref < = 10 years)	2 (1–5)	3 (1–4)	2.65 (1.41—4.99)	0.003
Men (ref Women)	2 (1–4)	2 (1.25–3)	0.96 (0.56—1.66)	0.9
*Scoring direction: 1 (less often)—5 (more often)*				
*OR* > *1 means more likely to have a higher score*				
*Likelihood ratio test (full model compared to intercept-only), x*^*2*^*(3)* = *48.1, p* < *0.001*				
***1.7 Do you feel you have been treating more or fewer patients with SMR over during your time in EMS?***				
ALS (ref BLS)	4 (4–5)	4 (3–4)	2.93 (1.58—5.43)	< .001
> 10 years exp. (ref < = 10 years)	4 (4–5)	4 (3–4)	1.33 (0.74—2.41)	0.3
Men (ref Women)	4 (3–4.5)	4 (3–4)	1.16 (0.66—2.05)	0.6
*Scoring direction: 1 (more)—5 (fewer)*				
*OR* > *1 means more likely to have a higher score*				
*Likelihood ratio test (full model compared to intercept-only), x*^*2*^*(3)* = *21.0, p* < *0.001*				
1. All scores expressed as median (interquartile range)				

*OR,* odds ratio; *MOI,* mechanism of injury; *ALS,* advanced life support; *BLS,* basic life support; *SMR,* spinal motion restriction

### Inter-rater reliability of judging traumatic MOIs

Section 4 of the survey evaluated participants’ agreement on categorizing a traumatic MOI as having the potential to cause a spine injury or not. Respondents showed fair agreement overall: k = 0.31 (95%CI: 0.08–0.48). Table 6 details agreement among each demographic sub-group and among all participants when evaluating particular patient groups and MOI types. While agreement among sub-groups was similar, all respondents showed higher agreement in evaluating scenarios related to low-level falls (k = 0.43, 95%CI: 0.04–0.68). In contrast, agreement was no better than chance on questions related to geriatrics, pediatrics, assaults, and MVCs.

**Table 6:** Inter-rater reliability of evaluations of mechanisms of injury in a paramedic spine survey

Group	Fleiss' kappa (95% CI)
All raters, all questions	0.31 (0.09—0.49)
**Demographic trait**	
ALS	0.31 (0.08—0.51)
BLS	0.31 (0.10—0.47)
> 10 years’ experience	0.31 (0.09—0.49)
< = 10 years’ experience	0.31 (0.12—0.47)
Men	0.31 (0.09—0.49)
Women	0.34 (0.07—0.51)
**MOI type**	
Geriatrics	0.03 (-0.01—0.05)
Pediatrics	0.03 (-0.01—0.03)
Assaults	0.04 (-0.01—0.04)
MVCs	-0.01 (-0.02—0.01)
Falls	0.43 (0.04—0.68)

*ALS,* advanced life support; *BLS,* basic life support; *MOI,* mechanism of injury; *MVC,* motor vehicle crash

### Content analysis

Table 7 displays the category map of free-text responses to seven open-ended questions [41] (Additional file 3 lists sub-categories by question.) A common thread evident among all responses was abstracted as a main category: tension between SMR-as-directed and SMR-as-applied. This main category captures the competing imperatives of working within and according to protocol while at the same time recognizing the limitations of SMR and adapting treatment on a patient-by-patient basis to optimize care – adaptations that sometimes step outside or work at the edges of written guidelines. Two categories support and provide more detailed descriptions of this sentiment.

**Table 7:** Inductive qualitative content analysis of free-text responses in a paramedic spine survey, with illustrative quotations

MAIN CATEGORY	TENSION BETWEEN SMR-AS-DIRECTED AND SMR-AS-APPLIED	
Categories	Complications and solutions in the application of SMR	Conflicting influences on how to apply SMR
Sub-categories	SMR causes motion	Direction from protocols and guidelines
	Adverse effects of SMR	Training in the procedure and higher education
	Efforts to minimize patient movement	Past experience with difficult/unusual situations
	Suggested improvements	Knowledge of recent research
		Influence of workplace culture
Illustrative quotations	*• Patient discomfort with the c-collar seems lead to many cases of patients readjusting, pulling at, attempting to remove c-collar, leading to increased manipulation of the neck [The] “no-neck” [smallest] size seems to help with patient comfort and reduce this*	*• [Past practice] led to a vast number of unnecessarily boarded patients. Change in protocol and more leeway in critical decision-making during assessment led to improvement in this area*
		*• More experience means comfort in defending/rationalizing my choice for SMR….Less willingness to treat in a certain way because “it’s always been that way.”*
	*• I’ve grown tired of fighting with people who are intoxicated, combative, *etc*., and … think I can make a case that not wrestling with someone and allowing them to not be immobilized is safer for them than wrestling with someone I suspect is truly injured*	
		*• More research done showing many adverse effects*
		*• [There is now] less fear in the workplace around disciplinary action towards not utilizing SMR*

The first category encompasses complications and solutions in the application of SMR. This category includes observations that SMR sometimes causes motion and knowledge of its adverse effects, as well as work-arounds and suggestions for improvement. Respondents frequently described SMR as less than effective, observing that that treatment devices aggravate almost all patients, and especially those who are anxious or agitated. One response describes this scenario and provides a justification for not treating a patient altogether:

*I’ve grown tired of fighting with people who are intoxicated, combative, etc., and ... think I can make a case that not wrestling with someone and allowing them to not be immobilized is safer for them than wrestling with someone I suspect is truly injured.*

Also citing the adverse effects of treatment, responses detailed additional strategies to minimize movement. These included consciously under-sizing cervical collars, allowing alternative positioning, and (in two cases) not treating when indicated by protocol. Suggested improvements included using a soft cervical collar (as opposed to rigid), allowing patients to self-extricate from vehicles, options for sedation, removing the long backboard for transport, and the ability to identify low-risk patients as candidates for further assessment without applying any restraining devices.

The second category summarizes conflicting influences on how to apply SMR. On one hand, participants recognized that they follow their training and work according to protocols and written guidelines. Some observed that protocols had been updated, advanced education had improved their understanding, and more ongoing training would improve care. At the same time, participants outlined a variety of alternative, sometimes contradictory, influences on their practice. These include past experiences with challenging situations not imagined in guidelines, familiarity with recent research highlighting the limitations of SMR, and differing standards in other jurisdictions. Notably, some respondents described a silent evolution of workplace standards away from strict protocol adherence with possible punishment for deviation and towards a culture with less emphasis on SMR. Whereas the prior approach to potential spine injuries was characterized as “gross over-caution,” participants described unwritten “employer expectation changes” and “less fear in the workplace around disciplinary action towards not utilizing SMR” as contributors to an evolving standard.

## Discussion

The results of this survey portray paramedic attitudes towards prehospital SMR as nuanced and complex. Although participants in this survey report that they generally follow relevant protocols, detailed responses illustrate many ways in which providers balance protocol adherence with attempts to optimize care. Most notably, responses demonstrate broad skepticism towards the value of SMR (raw data, Table 2). Particularly among ALS providers, rating responses and analyses indicate strongly that SMR is seen as less important than in the past and that they are treating fewer patients with SMR than before (Tables 4 and 5). Free-text responses expand on these findings and provide specific examples of ways in which paramedics navigate the practice environment and resolve the tension that arises when protocolized treatment does not match the clinical situation (Table 7).

While drawn from a single service, these findings are relevant for the treatment of potential spine injuries in general. These results inform current techniques or raise key questions for future study in three specific topic areas, whatever the status of local protocols. These topic areas include the connection between provider attitudes and treatment patterns, the application of selective immobilization protocols, and the use of specific devices, particularly cervical collars. Additionally, these findings can be placed in a broader context of paramedic decision-making, where they support including frontline providers in the process of guideline development and implementation.

First, the connection between the views of frontline providers and patterns of treatment has not been widely researched. Some studies have reported feelings of skepticism towards the value of SI or SMR among EMS personnel in a variety of settings, [1, 17, 18] and others have described differences in treatment before and after the transition to SMR, [19, 45] but the relationship between provider beliefs and treatment patterns deserves more exploration [46]. In the service being surveyed, there has been a decreasing trend in the number of treatments over the last decade [47]. It is not clear why SMR would be associated with fewer treatments or more missed injuries. One possible explanation is that SMR has done more than simply provide alternative assessment and treatment options: in considering the limitations of past practice, it has also shifted baseline assumptions of potential harms and benefits of treatment – implicitly granting permission for more widespread practice change. This view corresponds with earlier opinions of SI as over-used, un-necessary, or sub-optimal, [1, 17, 18] and respondents to this survey described exactly this shift in practice in terms of “less fear in the workplace…towards not utilizing SMR” and a move away from “gross over-treatment”. Whether this shift can be described as adequate correction or over-compensation remains to be seen. Further research should investigate patterns of care to determine not only trends in treatment but also clinical outcomes of patients with injuries who do not receive prehospital SMR at the local or individual level.

These survey results are also relevant for the use of selective immobilization protocols. A number of prior studies have documented the discrepancy between assessment findings and treatment provided, most often in the case of not applying devices when indicated [20–22]. While past research has noted substantial variation in how providers interpret MOIs, [21] and consequently whom they choose to assess, this area of decision-making has not been prospectively quantified. Section 4 of this survey was designed to assess exactly this. The results show that providers achieved only fair agreement on whether or not an MOI has the potential to cause a spine injury (Table 4). Although the magnitude of the kappa statistic can be affected by many factors and interpretations can be considered somewhat arbitrary, [48] it is not surprising that agreement among these MOIs, which were deliberately written to reflect marginal scenarios, appears relatively low. This finding serves as a reminder that spinal assessment protocols depend on a subjective decision to apply them, and, as prior research has shown, these decisions are both variable and clinically relevant for some groups [21]. While the risk of injury from a marginal MOI might be negligible for most patients, geriatrics appear to be over-represented among those with spine injuries and without prehospital treatment, often from scenarios thought to be low-risk [22, 49, 50]. Evaluations of assessment protocols should account for variation in how patients are identified as candidates and include an ability to audit cases where it was not applied.

The findings of this survey also relate to a third area of practice, the use of cervical collars. Within these results, 74% stated they apply a “no-neck” (the smallest among available sizes) most often, and 54% reported routinely measuring for collar size. Free-text responses commonly reported deliberately under-sizing collars to minimize patient discomfort and resulting movement. Neither self-reported collar-size distributions nor the rationale behind sizing choices made in the field has been described previously. In contrast, treatment guidelines that support collar use emphasize measuring and fitting the collar to the patient, using standard sizes that vary by millimeters [4, 7]. One study tested providers on their ability to apply a collar to a mannequin and judged that only 11% of participants were able to do so correctly [51]. These sources appear to base their determination of what is correct and proper on manufacturers’ guidelines and a small number of laboratory and cadaver studies that have investigated sizing [52–54]. Although these studies show increased restriction with fitted devices, their methodologies have limited connection to field conditions and their findings cannot be generalized to patient care. In contrast to guidelines that emphasize the importance of properly fitted collars, an increasing number of position statements cite sparse evidence of benefit and recommend variations on no use, judicious use, or soft alternatives [5, 8–10, 12]. Additionally, one recent study involving actual trauma patients found that patient movement depended more on patient behavior than the device applied [55]. In this context, it would be possible to interpret these survey results not as a signal of protocol non-compliance, but as frontline providers working within local requirements to balance the benefits and harms of treatment for their patients. It is unknown whether this practice tendency is unique to this service, but in the absence of any demonstrated benefit to sizing collars among available options, the notion of a what constitutes a properly fitted collar should be reconsidered.

As illustrated by the variety of approaches to something as superficially simple as putting on a cervical collar, the application of written protocols to varied circumstances requires substantial judgement from providers. The role of judgement has been acknowledged and studied particularly in the context of trauma-alert protocols, finding it to be a major factor [25, 26]. More detailed investigations of prehospital decision-making have consistently found that the process is not linear but complex and dynamic, and informed more by the experience, education, and tendencies of the provider than by written guidelines [26–28]. In both prehospital and emergency settings, protocols and clinical decision rules are seen to function as a cognitive scaffold and safety net for inexperienced providers instead of a practice template for all [27, 28, 30]. This understanding would see a gap between protocol and practice not as an issue of non-compliance or inadequate education, but as the inevitable result of applying linear tools to a complex, non-linear environment [30]. Although not previously applied to prehospital spinal care, this view provides a persuasive interpretative context for the attitudes and behaviors recorded in this study; it also supports the inclusion of provider judgement within treatment guidelines as well as end-user input into future revisions [25].

### Limitations

The results of this survey reflect the self-reported views of paramedics in one service at one time. It is unknown whether these results are generalizable to other services or other jurisdictions. Local SMR protocols vary, and not all questions and responses recorded here will be relevant to other agencies. Among participants, ALS providers were over-represented as compared to the composition of the service as a whole; it’s unknown how this response pattern might bias results. Among the submitted surveys, not all were complete (although the number excluded was relatively small).

## Conclusion

This survey reports prehospital providers’ beliefs, observations, and practices related to prehospital spinal care after the implementation of SMR. These results support continued research in several areas, including the assessment of treatment outcomes after SMR implementation, the application and execution of prehospital selective immobilization protocols, and the effectiveness of procedures and devices in field use. Although there is widespread agreement in the overall goal of reducing motion among potentially spine-injured patients, prehospital guidelines and protocols continue to show substantial variation. As standards evolve, input from frontline providers that reflects the practical realities of care in local circumstances should help shape future guidelines.

## Supplementary Information

**Additional file 1.** Survey Paramedic attitudes towards prehospital treatment of potential spine injuries

**Additional file 2. **Raw Data

**Additional file 3.** Free-text responses by question

## Data Availability

Portions of raw data are available in additional files. Remaining data are available from the corresponding author on reasonable request.

## Abbreviations

SMR: Spinal motion restriction
EMS: Emergency medical services
MOI: Mechanism of injury
EFA: Exploratory factor analysis
BLS: Basic life support
ALS: Advanced life support
OR: Odds ratio
EMTs: Emergency medical technicians
SI: Spinal immobilization
CROSS: Checklist for Reporting Of Survey Studies
IQR: Interquartile range
MVC: Motor vehicle crash
WFPS: Winnipeg Fire Paramedic Service
