# Supplementary material for: Paramedic attitudes towards prehospital spinal care: a cross-sectional survey
Source: BMC Emerg Med. 2022 Sep 20;22:162. doi: 10.1186/s12873-022-00717-2 (PMC9487099; doi:10.1186/s12873-022-00717-2)
Supplement: Supplementary file 1 — Additional file 1. Survey Paramedic attitudes towards prehospital treatment of potential spine injuries [file 12873_2022_717_MOESM1_ESM.pdf]

Survey

Paramedic attitudes towards prehospital treatment of potential spine injuries

1. Please confirm you are a licensed paramedic practicing with the [redacted] Service. This survey is open only to current [redacted] paramedics.
    a. I am a licensed paramedic with the [redacted] Service
    b. I am not a licensed paramedic with the [redacted] Service and will exit the survey.

2. What is your current qualification level / role?
    a. PCP/FFPCP
    b. ICP
    c. ACP/ACP-P
    d. DC/TO/PCPO

3. Please enter the number of years you have been practicing as a paramedic (as a number, rounded to the nearest year). Enter 0 if you are not a practicing paramedic.
    a. [free text for number]

4. Age [free text for number]

5. Gender
    a. Woman
    b. Man
    c. Transgender
    d. Non-binary/non-conforming
    e. Prefer not to respond

Section 1:
General Attitudes

This survey investigates paramedics' attitudes and practice around the use of spinal precautions. Formerly known as "spinal immobilization", these practices are more generally referred to as spinal motion restriction (SMR). This survey will use the term "SMR" to refer to all treatment (collar-only or board-and-collar) and "Spinal Immobilization" only when specifically referring to the intervention tab in the ePCR.

1.1 In your opinion, how effectively does SMR as currently practiced limit patient motion?
<- Not at all effectively … Very effectively ->

1.2 In your estimation, how often have you observed SMR *ineffectively* limit motion or cause more motion than no treatment or alternatives?
<- Very infrequently … Very frequently ->

1.3 Among patients at risk for spine injury and in SMR, how often do you observe patient motion that you feel could potentially cause further harm to their spine?
<- Very infrequently … Very frequently ->

1.4 In your opinion, could your service's current SMR protocols be changed to more effectively limit motion?
<- No, not at all … Yes, very much so ->

1.5 If your service's SMR could be improved, how would you like to change it (check all that apply)?

a. No change
b. Different assessment protocol / more leeway in choosing when to apply SMR
c. More options in terms of devices / patient positioning
d. Option not to apply any devices (including a cervical collar)
e. Other (free text)

1.6 If your service's SMR could be improved, which patient groups, if any, would benefit from modified or special treatment. (check all that apply)?

a. None
b. Geriatrics
c. Pediatrics
d. Intoxicated
e. Agitated / combative
f. Other (free text)

1.7 Do you feel you have been treating fewer or more patients with SMR over during your time in EMS?
<- Many fewer … Many more ->

1.8 Do you feel SMR is seen as less or more important than it was in the past?
<- Much less … Much more ->

1.9 If you feel there is a reason for a change in your practice over time, please explain: (short answer)

Section 2:
Specific Attitudes

2.1 In your opinion, how effectively does a cervical collar restrict head motion in a potentially spine-injured patient?

<- Not at all effectively … Very effectively ->

2.2 How often have you observed complications of a cervical collar resulting in more patient movement than no treatment or alternative / improvised treatment.

<- Very infrequently … Very frequently ->

2.3 Among patients at risk for spine injury and in a cervical collar, how often do you observe patient movement that you feel could potentially cause further harm to their spine?

<- Very infrequently … Very frequently ->

2.4 What size cervical collar do you apply most often?

a. No neck
b. Short
c. Medium
d. Tall
e. Improvised

2.5 How often do you measure a patient's neck to select a cervical collar?

<- Very infrequently … Very frequently ->

2.6 If you do not very frequently/always measure, which explanation best explains why:

a. Not applicable: I very frequently/always measure
b. It doesn't make a difference / I don't care
c. I would like to, but don't have time / don't have different sized collars
d. I intentionally apply shorter collars for patient comfort
e. Other (free text)

2.7 When treating a patient with isolated penetrating trauma to the head, neck, or torso, how often do you apply spinal precautions.

<- Very infrequently … Very frequently ->

2.8 When treating a patient with a known or suspected traumatic brain injury for whom spinal precautions are also indicated, how often do you loosen or remove a cervical collar?

<- Very infrequently … Very frequently ->

2.9 If a standard collar does not seem appropriate for a patient (due to usual anatomy or extremes of age, for example), how often would you apply an improvised collar such as a towel roll?

<- Very infrequently … Very frequently ->

2.10 If a patient is actively fighting against treatment devices (c-collar, straps, head blocks), how often would you remove, loosen, or modify the devices?

<- Very infrequently … Very frequently ->

2.11 For patients who require spinal precautions but are actively vomiting, how often would you secure them in the lateral / recovery position as opposed to rolling them each time they vomit?

<- Very infrequently … Very frequently ->

2.12 How often do you secure an SMR patient in a position other than supine?

<- Very infrequently … Very frequently ->

2.13 If you do not very frequently/always position your patient supine, which other position do you use most frequently?

a. Not applicable: always supine
b. Lateral
c. Semi-Fowler's
d. Sitting
e. Other

Section 3:
Spinal assessment protocols

3.1 In general, how often do you follow the criteria of the c-spine management protocol to determine the need for SMR in the setting of trauma with the potential for spine injury?
<- Very infrequently … Very frequently ->

3.2 In general and in your opinion, would you rate your service's criteria for determining the need for spinal precaution as not restrictive enough (patients left untreated who need it) or too restrictive (too many patients treated who do not need it)?
<- Not restrictive enough … Too restrictive ->

3.3 Do you ever use spinal precautions when they are not indicated by protocol?
<- Very infrequently … Very frequently ->

3.4 In which cases would you opt to use spinal precautions when they are not indicated by protocol (check all that apply)?
   a. Not applicable: I never use them when not indicated by protocol
   b. Believe they are necessary / protocol is not sufficient
   c. Instructed by senior provider to do so
   d. Worried about liability / opinion of receiving facility
   e. Precautions already placed by other providers
   f. Other (free text)

3.5 Do you ever not use spinal precautions when they are indicated by protocol for reasons other than refusal?
<- Very infrequently … Very frequently ->

3.6 In which cases would you opt not to use spinal precautions when indicated by protocol (other than cases of refusal, check all that apply)?
   a. Not applicable: I always use them when indicated by protocol
   b. Don't believe they are necessary / protocol is too restrictive
   c. Could potentially cause harm to this patient
   d. Find an alternative technique.
   e. Other (free text)

Section 4:
Judging MOIs

WFPS documentation uses a closed-call rule to prompt users to consider the c-spine management protocol in all trauma cases. The first field asks providers to categorize the mechanism of injury as either having no potential for spine injury (allowing users to exit the protocol) or the potential for spine injury (requiring assessment).

How would you document the following mechanisms of injury?

4.1 An adult, unrestrained car passenger, ejected after a crash at 100km/hr.
   a. Trauma w/ No Potential for C-Spine Injury
   b. Trauma w/ Potential for C-Spine Injury

4.2 Young adult, playing soccer, rolls over on ankle. No trauma to head. No contact with other players.
   a. Trauma w/ No Potential for C-Spine Injury
   b. Trauma w/ Potential for C-Spine Injury

4.3 Adult, assaulted. Punched in the face. No weapons used. Fell to the ground.
   a. Trauma w/ No Potential for C-Spine Injury
   b. Trauma w/ Potential for C-Spine Injury

4.4 Adult, tripped while walking. Fell on out-stretched arm. Complaining of shoulder pain. No trauma to the head.
   a. Trauma w/ No Potential for C-Spine Injury
   b. Trauma w/ Potential for C-Spine Injury

4.5 Adult, tripped coming down stairs. Fell to the ground from one step.
   a. Trauma w/ No Potential for C-Spine Injury
   b. Trauma w/ Potential for C-Spine Injury

4.6 Adult, fall from standing. Laceration to the face. No loss of consciousness.
   a. Trauma w/ No Potential for C-Spine Injury
   b. Trauma w/ Potential for C-Spine Injury

4.7 Elderly adult (>65). Fall from standing. Laceration to the face. No loss of consciousness.
   a. Trauma w/ No Potential for C-Spine Injury
   b. Trauma w/ Potential for C-Spine Injury

4.8 Elderly adult (>65), assaulted. Punched in the face. No weapons. Fell to the ground.
   a. Trauma w/ No Potential for C-Spine Injury
   b. Trauma w/ Potential for C-Spine Injury

4.9 Adult, restrained driver, MVC while turning left. Hit by a vehicle travelling 40 - 50 km/hr on the passenger side. Moderate damage at point of impact. Front air-bags deployed. Windshield intact.
   a. Trauma w/ No Potential for C-Spine Injury
   b. Trauma w/ Potential for C-Spine Injury

4.10 Child (7 years old), restrained on a booster seat on the driver's side, rear. MVC while turning left. Hit by a vehicle travelling 40 - 50 km/hr on the passenger side. Moderate damage at point of impact. Front air-bags deployed. Windshield intact.
   a. Trauma w/ No Potential for C-Spine Injury
   b. Trauma w/ Potential for C-Spine Injury

4.11 Elderly adult (>65). Syncopal episode. Fall from standing.
   a. Trauma w/ No Potential for C-Spine Injury
   b. Trauma w/ Potential for C-Spine Injury

4.12 Child (8 years old), fall from a slide onto grass, 2 meters. Hit head. Unknown if there was a loss of consciousness.
   a. Trauma w/ No Potential for C-Spine Injury
   b. Trauma w/ Potential for C-Spine Injury

4.13 In general, if you feel that a mechanism of injury is uncertain for its potential to cause a spine injury, what do you do?
   a. Choose "Trauma w/ No Potential for C-Spine Injury" and assess for and treat other problems.
   b. Choose "Trauma w/ Potential for C-Spine Injury", assess by protocol and treat according to assessment findings.
   c. Defer to your partner or another provider
   d. Other (free text)

Section 4: Documentation

The following questions were included in the survey, but have been analyzed separately and are not considered in this paper.

4.14 When you have treated your patient with spinal precautions, how frequently do you use the "Spinal Immobilization" intervention in the ePCR to document it?

1-very infrequently/never, 2-infrequently 3-about half the time, 4-frequently, 5-very frequently/always

4.15 When using the "Spinal Immobilization" intervention, how frequently would you say the intervention is time-stamped within 15 minutes of when it actually occurred?

1-very infrequently/never, 2-infrequently 3-about half the time, 4-frequently, 5-very frequently/always

The remaining questions refer to this scenario:

You are called by police to assess and adult male who was assaulted. On arrival, you find a grown man sitting on the curb. He shows signs of being intoxicated and has an empty bottle of alcohol in his pocket. You see a fresh laceration on his forehead that is oozing blood. When you ask the patient if anything is wrong, he says: "I'm short of breath. I can't breathe." The patient has no other complaints, no other physical findings, no signs of drug use other than alcohol, and vital signs all within normal ranges.

4.16 How would you document this patient's Chief Complaint?

a. Substance misuse
b. Assault OR head injury OR head pain
c. Shortness of breath
d. Other

4.17 How would you document your Primary Impression for this patient?

a. Substance misuse (alcohol)
b. Any Trauma impression
c. Respiratory (other respiratory problem)
d. Other

4.18 How would you document this patient's initial CTAS Category and Chief Complaint?

a. Respiratory – Shortness of Breath
b. Neurology – Head Injury OR Trauma – any complaint
c. Substance Misuse – Substance Abuse / Intoxication
d. Other
