# Supplementary material for: Paramedic attitudes towards prehospital spinal care: a cross-sectional survey
Source: BMC Emerg Med. 2022 Sep 20;22:162. doi: 10.1186/s12873-022-00717-2 (PMC9487099; doi:10.1186/s12873-022-00717-2)

Supplemental File 2
Raw Data: Section 1

1.1 In your opinion, how effectively does SMR as currently practiced limit patient motion?

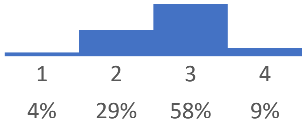

<- Not at all effectively … Very effectively ->

1.2 In your estimation, how often have you observed SMR ineffectively limit motion or cause more motion than no treatment or alternatives?

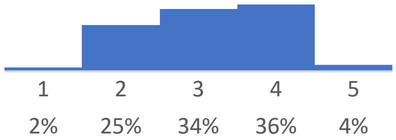

<- Very infrequently … Very frequently ->

1.3 Among patients at risk for spine injury and in SMR, how often do you observe patient motion that you feel could potentially cause further harm to their spine?

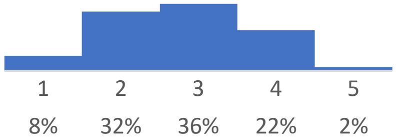

<- Very infrequently … Very frequently ->

1.4 In your opinion, could your service's current SMR protocols be changed to more effectively limit motion?

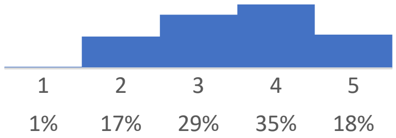

<- No, not at all … Yes, very much so ->

Supplemental File 2
Raw Data: Section 1

1.5 If your service's SMR could be improved, how would you like to change it (check all that apply)?

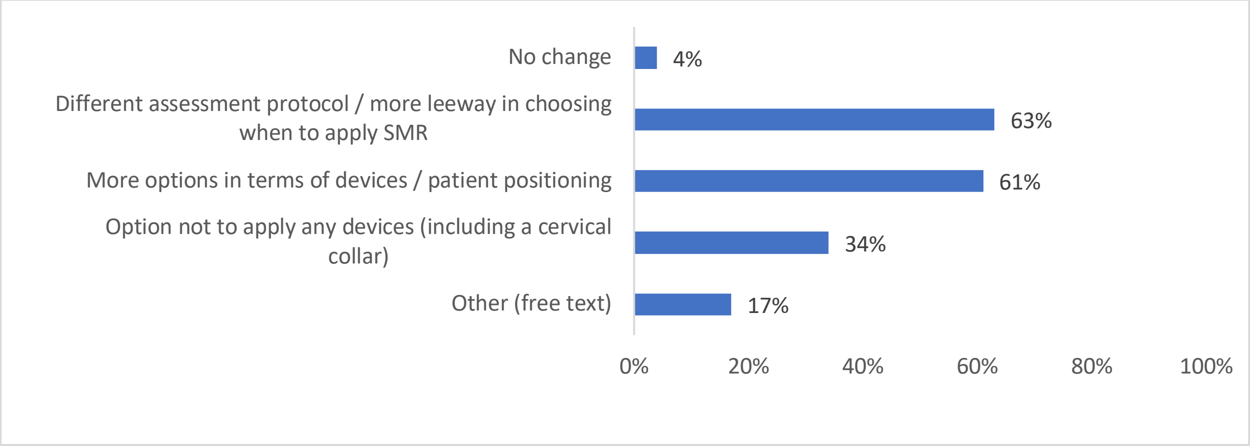

1.6 If your service's SMR could be improved, which patient groups, if any, would benefit from modified or special treatment. (check all that apply)?

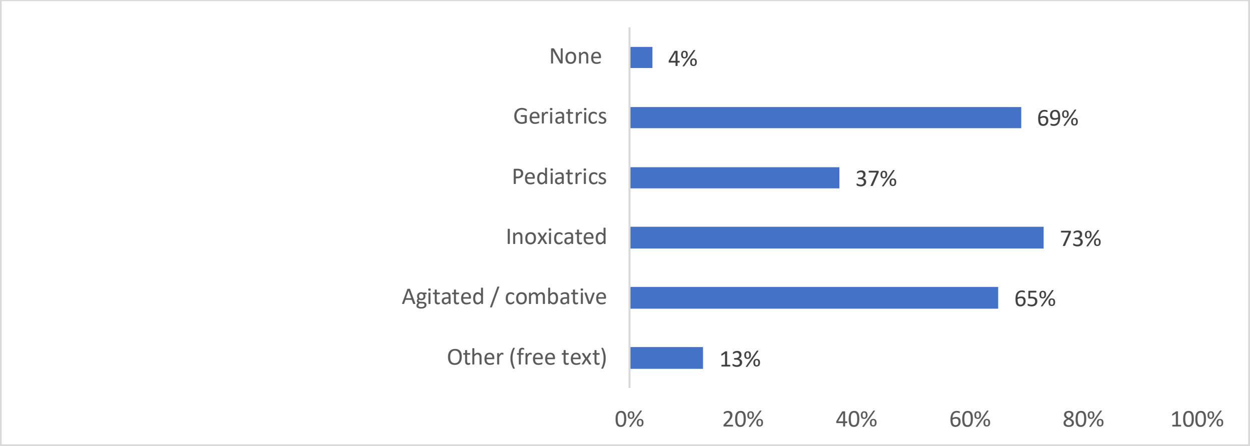

1.7 Do you feel you have been treating fewer or more patients with SMR over during your time in EMS?

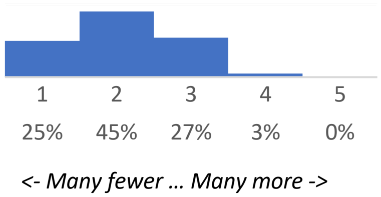

1.8 Do you feel SMR is seen as less or more important than it was in the past?

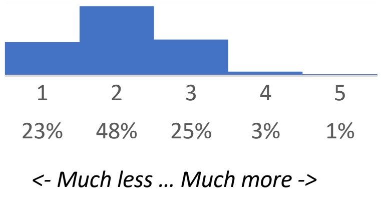

Supplemental File 2
Raw Data: Section 2

2.1 In your opinion, how effectively does a cervical collar restrict head motion in a potentially spine-injured patient?

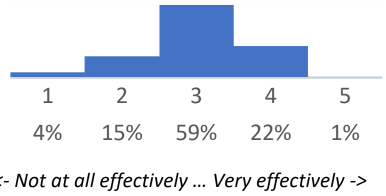

2.2 How often have you observed complications of a cervical collar resulting in more patient movement than no treatment or alternative / improvised treatment.

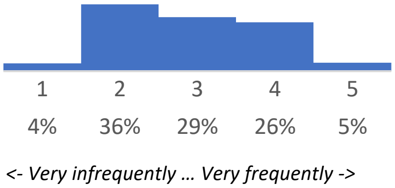

2.3 Among patients at risk for spine injury and in a cervical collar, how often do you observe patient movement that you feel could potentially cause further harm to their spine?

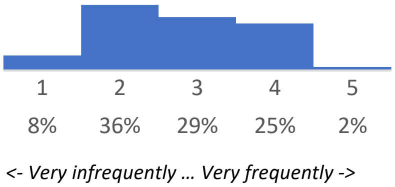

2.4 What size cervical collar do you apply most often?

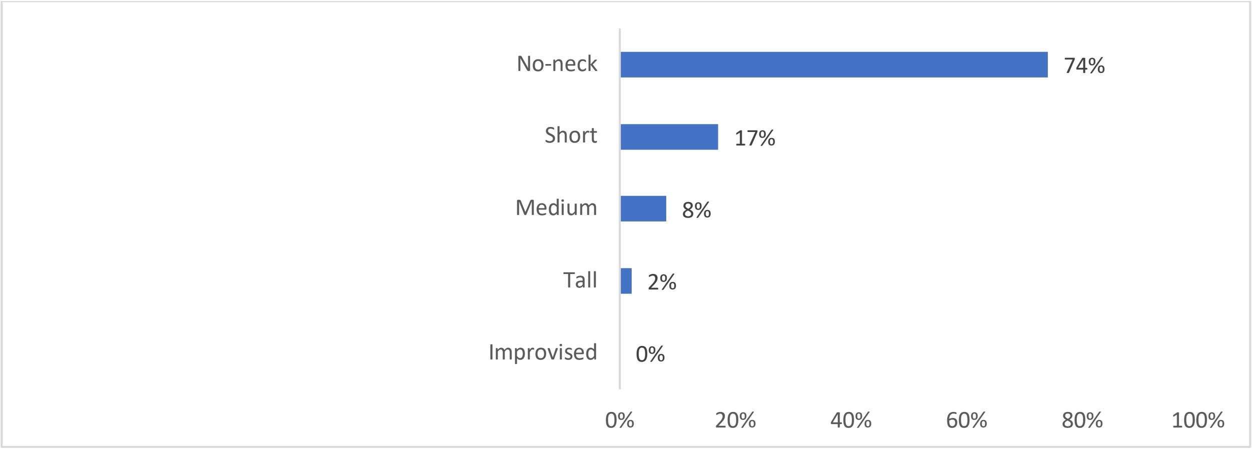

2.5 How often do you measure a patient's neck to select a cervical collar?

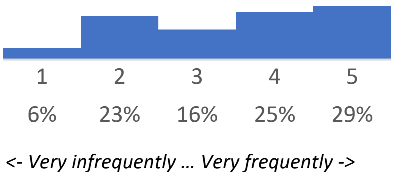

Supplemental File 2
Raw Data: Section 2

2.6 If you do not very frequently/always measure, which explanation best explains why:

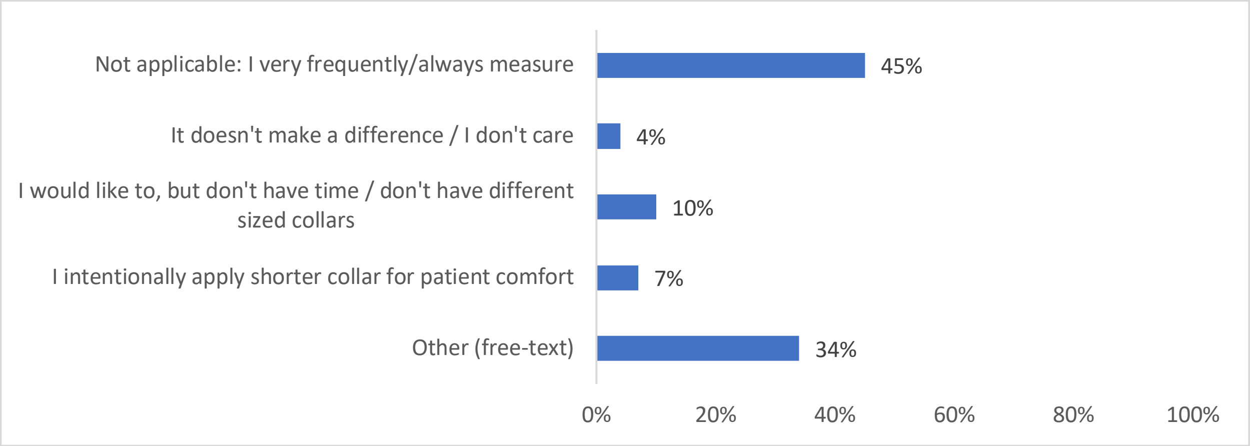

2.7 When treating a patient with isolated penetrating trauma to the head, neck, or torso, how often do you apply spinal precautions.

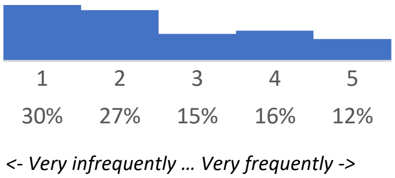

2.8 When treating a patient with a known or suspected traumatic brain injury for whom spinal precautions are also indicated, how often do you loosen or remove a cervical collar?

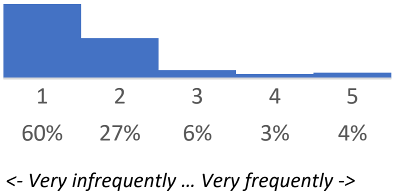

2.9 If a standard collar does not seem appropriate for a patient (due to usual anatomy or extremes of age, for example), how often would you apply an improvised collar such as a towel roll?

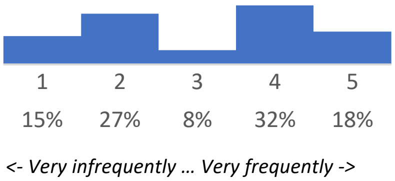

Supplemental File 2
Raw Data: Section 2

2.10 If a patient is actively fighting against treatment devices (c-collar, straps, head blocks), how often would you remove, loosen, or modify the devices?

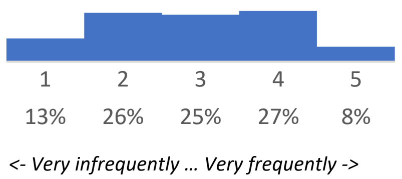

2.11 For patients who require spinal precautions but are actively vomiting, how often would you secure them in the lateral / recovery position as opposed to rolling them each time they vomit?

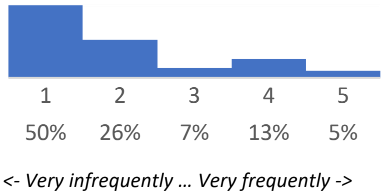

2.12 How often do you secure an SMR patient in a position other than supine?

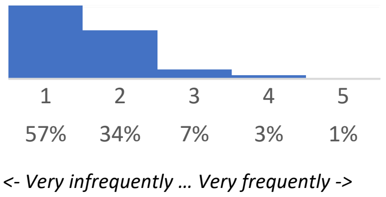

2.13 If you do not very frequently/always position your patient supine, which other position do you use most frequently?

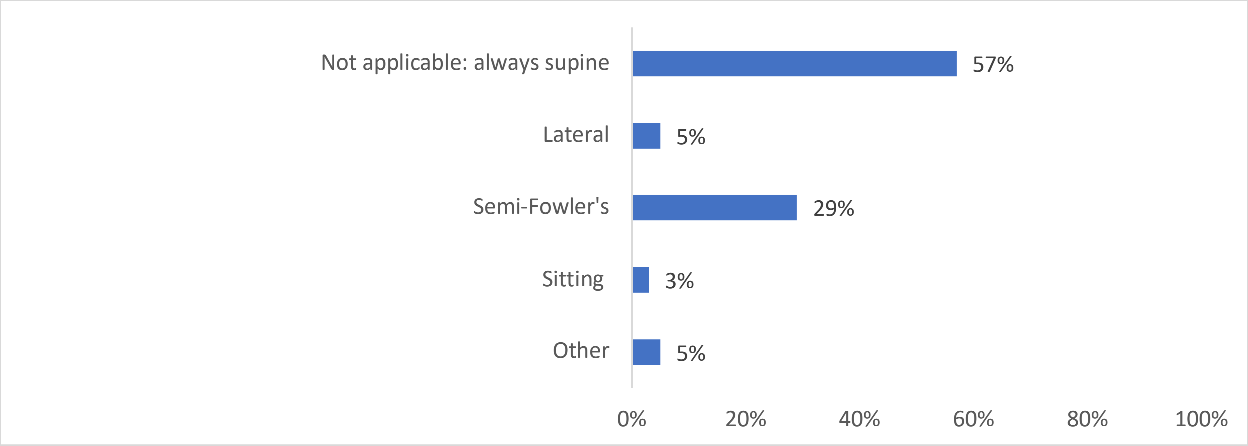

Supplemental File 2
Raw Data: Section 3

3.1 In general, how often do you follow the criteria of the c-spine management protocol to determine the need for SMR in the setting of trauma with the potential for spine injury?

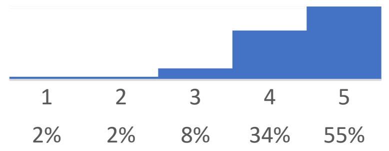

<- Very infrequently … Very frequently ->

3.2 In general and in your opinion, would you rate your service's criteria for determining the need for spinal precaution as not restrictive enough (patients left untreated who need it) or too restrictive (too many patients treated who do not need it)?

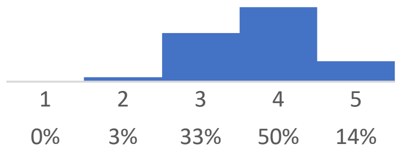

<- Not restrictive enough … Too restrictive ->

3.3 Do you ever use spinal precautions when they are not indicated by protocol?

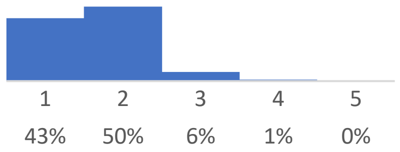

<- Very infrequently … Very frequently ->

3.4 In which cases would you opt to use spinal precautions when they are not indicated by protocol (check all that apply)?

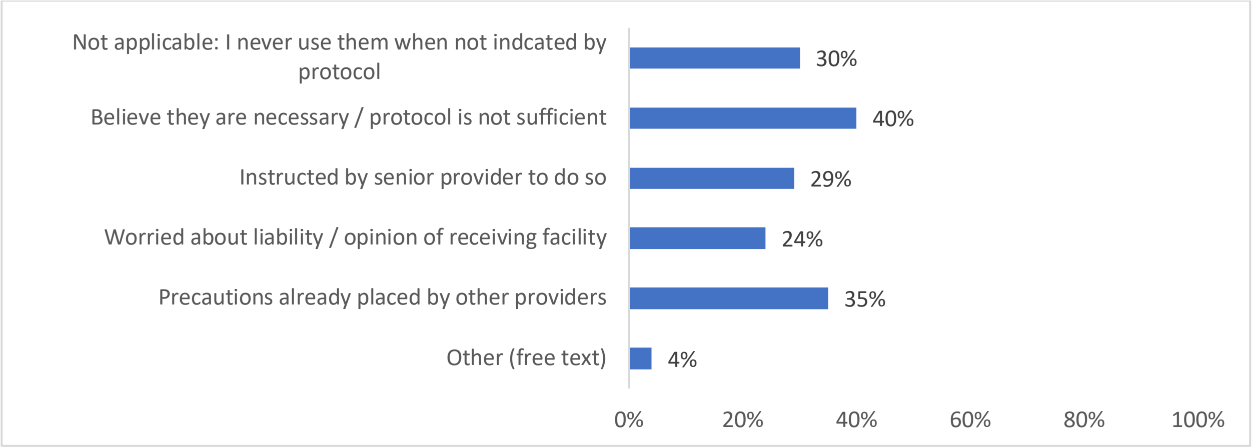

Supplemental File 2
Raw Data: Section 3

3.5 Do you ever NOT use spinal precautions when they are indicated by protocol for reasons other than refusal?

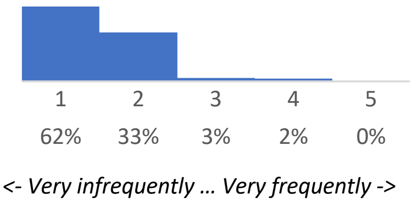

3.6 In which cases would you opt not to use spinal precautions when indicated by protocol (other than cases of refusal, check all that apply)?

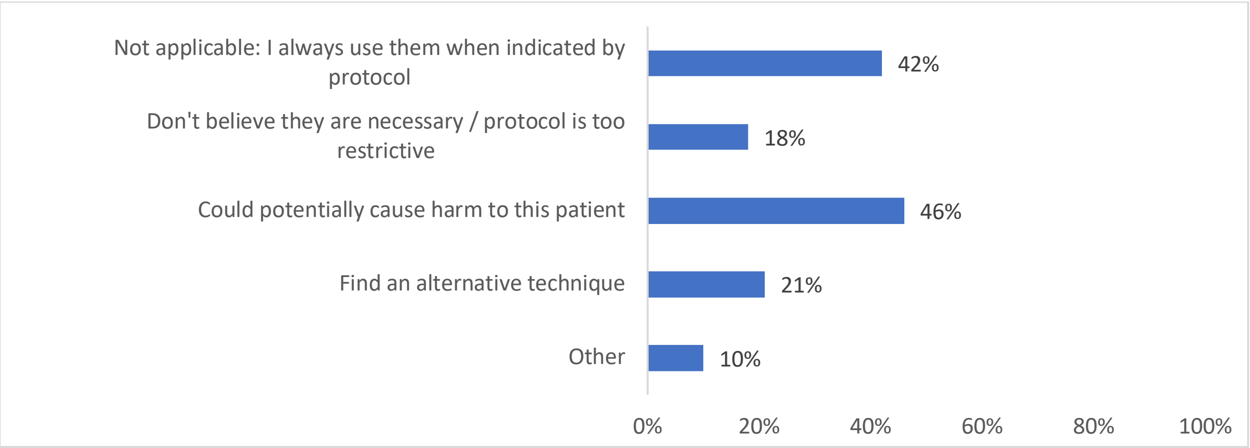

Supplemental File 2
Raw Data: Section 4

4.1 An adult, unrestrained car passenger, ejected after a crash at 100km/hr.

Potential for spine inj.	100%
No potential for spine inj.	0%

4.2 Young adult, playing soccer, rolls over on ankle. No trauma to head. No contact with other players.

Potential for spine inj.	1%
No potential for spine inj.	99%

4.3 Adult, assaulted. Punched in the face. No weapons used. Fell to the ground.

Potential for spine inj.	53%
No potential for spine inj.	47%

4.4 Adult, tripped while walking. Fell on out-stretched arm. Complaining of shoulder pain. No trauma to the head.

Potential for spine inj.	3%
No potential for spine inj.	97%

4.5 Adult, tripped coming down stairs. Fell to the ground from one step.

Potential for spine inj.	25%
No potential for spine inj.	75%

4.6 Adult, fall from standing. Laceration to the face. No loss of consciousness.

Potential for spine inj.	19%
No potential for spine inj.	81%

4.7 Elderly adult (>65). Fall from standing. Laceration to the face. No loss of consciousness.

Potential for spine inj.	61%
No potential for spine inj.	39%

4.8 Elderly adult (>65), assaulted. Punched in the face. No weapons. Fell to the ground.

Potential for spine inj.	74%
No potential for spine inj.	26%

4.9 Adult, restrained driver, MVC while turning left. Hit by a vehicle travelling 40 - 50 km/hr on the passenger side. Moderate damage at point of impact. Front air-bags deployed. Windshield intact.

Potential for spine inj.	79%
No potential for spine inj.	21%

4.10 Child (7 years old), restrained on a booster seat on the driver's side, rear. MVC while turning left. Hit by a vehicle travelling 40 - 50 km/hr on the passenger side. Moderate damage at point of impact. Front air-bags deployed. Windshield intact.

Potential for spine inj.	79%
No potential for spine inj.	21%

4.11 Elderly adult (>65). Syncopal episode. Fall from standing.

Potential for spine inj.	51%
No potential for spine inj.	49%

4.12 Child (8 years old), fall from a slide onto grass, 2 meters. Hit head. Unknown if there was a loss of consciousness.

Potential for spine inj.	91%
No potential for spine inj.	9%

Supplemental File 2
Raw Data: Section 4

4.13 In general, if you feel that a mechanism of injury is uncertain for its potential to cause a spine injury, what do you do?

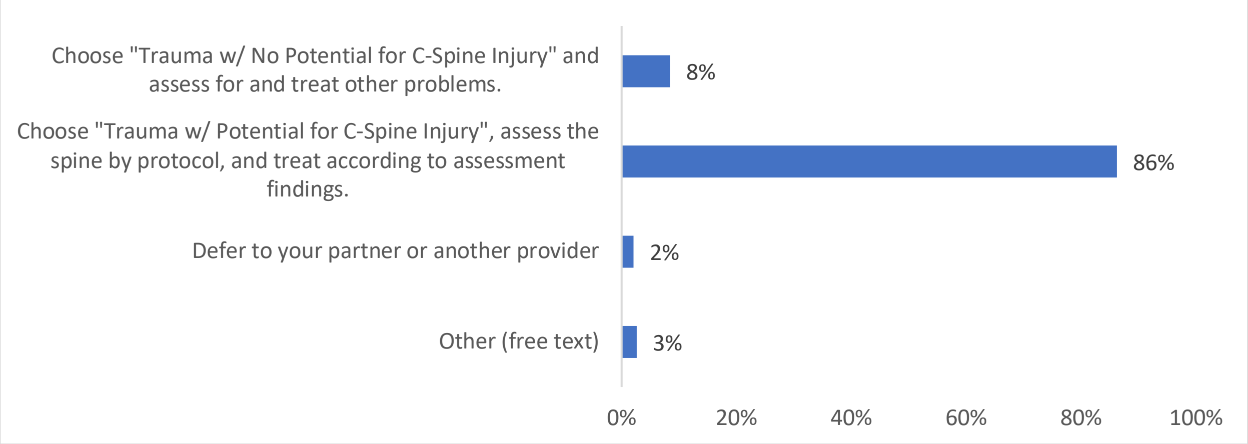

Supplement: Supplementary file 2 — Additional file 2. Raw Data [file 12873_2022_717_MOESM2_ESM.pdf]
