# Supplementary material for: Paramedic attitudes towards prehospital spinal care: a cross-sectional survey
Source: BMC Emerg Med. 2022 Sep 20;22:162. doi: 10.1186/s12873-022-00717-2 (PMC9487099; doi:10.1186/s12873-022-00717-2)
Supplement: Supplementary file 3 — Additional file 3. Free-text responses by question [file 12873_2022_717_MOESM3_ESM.pdf]

Free-text responses by question

Number of entries (% of total respondents)	Sub-categories	Count of mentions (% of responses to question)[1]	Illustrative Quotation(s)[2]
**1.5: If your service's SMR could be improved, how would you like to change it?**			
34 (17)	Suggested improvements	17 (50)	*Use of a lanyard to indicate c-spine not 'cleared' in patients requiring further assessment but not displaying any signs of spinal cord injury or severe midline neck pain*
	SMR causes motion	11 (32)	*Hard collars only seem to provide discomfort [to] patients. Repeatedly moving and fighting the collar results in more movement, rendering the whole process redundant.*
	Adverse effects of SMR	6 (18)	*[use] soft collars instead of hard [because of increasing] ICP secondary to compressed external jugular veins*
	Knowledge of recent research	6 (18)	*the current research as well as provincial protocols shows that restricting patients on a [long spine board] has no benefit after the damage is done*
	Training in the procedure and higher eduction	7 (21)	*Continuing education for members to review current and best practice*
**1.6 If your service's SMR could be improved, which patient groups, if any, would benefit from modified or special treatment? Option for "other" with free text**			
26 (13)	Past experience with difficult / unusual situations	19 (73)	**Bariatrics; *geriatrics; *wheelchair-dependent; *pre-existing conditions eg scoliosis; *sports collisions.*
	SMR causes motion	7 (27)	*Bariatric/obese; those cannot tolerate laying supine*
	Adverse effects of SMR	3 (12)	*It's very painful for geriatrics and causes further issues such as pressure sores*

Free-text responses by question

1.9: If you feel there is a reason for a change in your practice over time, please explain: (short answer)

90 (46)	Direction from protocols and guidelines	37 (41)	*When the protocols changed for SMR, it was much more of a relief to not have a patient lying on a longboard for several hours on scene and at hospital causing lots of increased pain and discomfort.*
	Knowledge of recent research	34 (38)	*More research done showing many adverse effects of being on a backboard for lengthy periods of time.*<br>** Increased research and evidence showing that full SMR was often over utilized and caused further harm (pressure wounds, anxiety) and the potential for more spinal motion than alternative solutions.*
	Efforts to minimize patient movement	23 (26)	*I've grown tired of fighting with people who are intoxicated, combative, etc., and in the interest of non malfeasance think I can make a case that not wrestling with someone and allowing them to not be immobilized is safer for them than wrestling with someone I suspect is truly injured.*
	Past experience with difficult / unusual situations	17 (19)	*My experience has shown that immediately following SMR patient's can be cooperative but that quickly changes to agitated/uncomfortable and uncooperative. Causing more stress to the patient and their injuries (if any).*<br>**More experience means comfort in defending/rationalizing my choice for SMR.*
	Training in the procedure and higher eduction	14 (16)	*Also through my years I have gone from being a [BLS provider] to a licensed [ALS provider] so feel my assessment skills, knowledge and judgement have improved*
	Influence of workplace culture	12 (13)	**Less fear in the workplace around disciplinary action towards not utilizing SMR.*<br>**[Past practice] led to a vast number of unnecessarily boarded patients. Change in protocol and more leeway in critical decision making during assessment led to improvement in this area*

2.6: If you do not very frequently/always measure [neck size for a cervical collar], which explanation best explains why (other free-text)?

63 (32)	Efforts to minimize patient movement	36 (57)	*Very rarely does the measurement provide any correlation with pts actual size. Hair/clothing, jaw size, weight all play into the comfort level of a pt in a cervical collar, If you place a collar on that is to small. it will provide more comfort to the patient and allow for you to leave in place to prevent you from having to take it out adjust and repeatedly causing more movement of a pt with a suspected spinal injury.*<br>** Even on tall pts with long necks, it seems that collars don't properly conform to the individual body types (chest,shoulder,back,clothing). No neck collars seem to immobilize the patient with the least amount of discomfort resulting in less movement of the pt. Even if a different sized collar would fit the PT better it is typically much more uncomfortable and the PT almost always ends up moving more. It appears the collar operates more so as a " conscious reminder" for the PT not to move rather than something that actually physically limits the movement.*
	Past experience with difficult / unusual situations	27 (43)	*Experience - better a estimating the size*
	Training in the procedure and higher eduction	2 (3)	*Need to be trained on how to properly measure.*
	Knowledge of recent research	1 (2)	*In my own research I have not been able to find any benefit to the treatment at all let alone to measuring.*

Free-text responses by question

3.4: In which cases would you opt to use spinal precautions when they are not indicated by protocol (other free text)?

8 (4)	[indications present]	4 (50)	*Alcohol or drug intoxication; unconscious/responsive*
	Past experience with difficult / unusual situations	4 (50)	*Other lower spinal injuries, and using clinical judgment*

3.6: In which cases would you opt NOT to use spinal precautions when indicated by protocol, other than refusal (other free text)?

17 (9)	SMR causes motion	16 (88)	**Agitated patient that is fighting SMR/ moving more than if there were less SMR*<br>**Geriatrics with kyphosis make a good example where c-collars are difficult to apply and I would use alternative immobilization techniques (such as towel rolls)*
	Efforts to minimize patient movement	2 (11)	*Some criteria met by protocol for SMR, but mild in severity (ie. Trauma in >65 but head trauma is minor, or distracting painful injury is not severe, with no midline neck pain) knowing that spine board will be very uncomfortable for patient and will cause suffering.*
	Past experience with difficult / unusual situations	2 (11)	*If it means that they will accept further care instead of a AMA [against medical advice] or refusal*

1. Not exclusive. Each response can apply to multiple sub-categories
2. Multiple quotations separated by an asterix (*)

SMR: Spinal Motion Restriction; ICP: intracranial pressure; MOI: mechanism of injury
Question 4.13 also had a free-text option but received no responses
